# Which are the duties and the limits of nursing in neonatal ventilation?

**DOI:** 10.1186/1824-7288-41-S1-A25

**Published:** 2015-09-24

**Authors:** Salvatore Muscolo

**Affiliations:** 1 Pediatric nurse, Neonatology and neonatal intensive care, Fondazione Ca’ Granda, Ospedale Maggiore Policlinico IRCCS Milano, Italy

## 

It's difficult to talk about nurse's autonomy in ventilation assistance, because is a medical prescription. However in accordance with the Code of ethics, the professional profile and law 42/99, nurses have decision and operative autonomy in nursing care, which is achieved through specific autonomous and complementary interventions of intellectual, technical-scientific, managerial, relational and educational nature [1-3]. The treatment of respiratory meets the need: help optimize gas exchange, reduce breathing exertion and promote healing process reducing hemodynamic interferences. These goals can be reached individualizing the treatment according to the pathophysiological features of the disease and the time evolution of single pathology.

Monitoring peripheral oxygen saturation is more suitable method in the ventilated preterm (<27 weeks) because transcutaneous oxygenation monitoring is not of routine use for lack of an adequate correlation with the blood gas and for highly sensitive skin.

Many studies suggest the prevention of lung damage and retinopathy of preterm concerning a prolonged hyperoxia by setting alarm limits in the event of administration of an oxygen concentration higher than 21%. Numerous clinical conditions, including the need for mechanical ventilation, can affect and change the brain oxygenation. The near-infrared ray spectrophotometry (NIRS) is a technique that allows non-invasive monitoring of oxygenation and cerebral hemodynamics. It provides a single quantitative parameter rSO2 (regional saturation of oxygen) as an index of tissue oxygenation [4].

Compared to the intubated newborn there is not a unique method and standardized anchorage of the endotracheal tube. The quality of the attachment can vary greatly depending on the choice of the tape and according to the method of taping adopted.

No recommendation exists in the literature about the method of taping: the most widely used systems include the use of one or two strips cut to Y or H. The adhesive tape, indisputably considered the system capable of guaranteeing the best results in terms of sealing, when applied with a taping system and encoded together with a hydrocolloid protective largely reduces the risk of injury[5-8].

The aspiration of the airways in infants should be based on careful clinical assessment and not on a routine basis. It recommended to avoid suctioning the nose but use saline drops instead, then suction the oropharynx [9].

Care posture is crucial because it promotes the stabilization of the neonatal functions and prevents bad posture [10].

It is recommended the use of ventilation systems with manual pressure control and delivered volumes in order to safeguard the delicate lung tissue.
